# Comparison of all-cause and malaria-specific mortality from two West African countries with different malaria transmission patterns

**DOI:** 10.1186/1475-2875-7-15

**Published:** 2008-01-18

**Authors:** Robert P Ndugwa, Heribert Ramroth, Olaf Müller, Momodou Jasseh, Ali Sié, Bocar Kouyaté, Brian Greenwood, Heiko Becher

**Affiliations:** 1 Department of Tropical Hygiene and Public Health, University of Heidelberg, Im Neuenheimer Feld 324, 69120 Heidelberg, Germany; 2 Medical Research Council Laboratories, Farafenni, The Gambia; 3 Centre de Recherché en Sante de Nouna (CRSN), BP 02, Nouna, Burkina Faso; 4 Centre National de Recherche et de la Formation sur le Paludisme, Ouagadougou, Burkina Faso; 5 London School of Hygiene and Tropical Medicine, London, UK

## Abstract

**Background:**

Malaria is a leading cause of death in children below five years of age in sub-Saharan Africa. All-cause and malaria-specific mortality rates for children under-five years old in a mesoendemic malaria area (The Gambia) were compared with those from a hyper/holoendemic area (Burkina Faso).

**Methods:**

Information on observed person-years (PY), deaths and cause of death was extracted from online search, using key words: "Africa, The Gambia, Burkina Faso, malaria, *Plasmodium falciparum*, mortality, child survival, morbidity". Missing person-years were estimated and all-cause and malaria-specific mortality were calculated as rates per 1,000 PY. Studies were classified as longitudinal/clinical studies or surveys/censuses. Linear regression was used to investigate mortality trends.

**Results:**

Overall, 39 and 18 longitudinal/clinical studies plus 10 and 15 surveys and censuses were identified for The Gambia and Burkina Faso respectively (1960–2004). Model-based estimates for under-five all-cause mortality rates show a decline from 1960 to 2000 in both countries (Burkina Faso: from 71.8 to 39.0), but more markedly in The Gambia (from 104.5 to 28.4). The weighted-average malaria-specific mortality rate per 1000 person-years for Burkina Faso (15.4, 95% CI: 13.0–18.3) was higher than that in The Gambia (9.5, 95% CI: 9.1–10.1). Malaria mortality rates did not decline over time in either country.

**Conclusion:**

Child mortality in both countries declined significantly in the period 1960 to 2004, possibly due to socio-economic development, improved health services and specific intervention projects. However, there was little decline in malaria mortality suggesting that there had been no major impact of malaria control programmes during this period. The difference in malaria mortality rates across countries points to significant differences in national disease control policies and/or disease transmission patterns.

## Background

Mortality rates in sub-Saharan Africa (SSA) remain very high, especially when compared to other regions of the world [1,2]. While mortality in many parts of the world has declined markedly in the last century, in SSA reductions have been less, although marked differences in trends between urban and rural areas are still observed with trends declining faster in urban areas [1,3-5]. A recent report from the World Health Organization estimates that each year there are about ten million deaths worldwide in children below five years of age [1]. The leading causes of death among children in this age-group are pneumonia, diarrhoea, malaria, and perinatal causes.

Approximately 90% of all malaria deaths in the world occur in Africa [1,6]. Major factors that contribute to this high mortality from malaria in Africa include poor access to health services, low quality of health services, and the increased resistance of malaria parasites to affordable first-line drugs such as chloroquine and sulphadoxine-pyrimethamine [7,8]. There is some evidence for a positive correlation between *Plasmodium falciparum *transmission intensity and malaria mortality but there are few data on this point and also such analysis is hampered by the difficulties in assessing causes of death [9-11]. Moreover, in regions where malaria is endemic, an association between observed all-cause mortality rates and malaria-specific mortality rates, especially among the children below five years of age would be expected. However, it is difficult to substantiate this association due to the scarcity of quality data from Africa on all-cause and in particular cause-specific mortality. Many surveys and a few censuses have been conducted in Africa since the early 1950s and these provide information on all-cause mortality rate but not on cause-specific mortality [12]. To address this gap, several demographic surveillance systems (DSS) have been established in developing countries where overall mortality, cause-specific mortality determined using the post-mortem questionnaire technique, morbidity, socio-economic status and migration can be studied [13].

In this study, data from surveys and censuses, as well as DSS data obtained from The Gambia and Burkina Faso were used to systematically compare all-cause and cause-specific mortality rates from under five children. Burkina Faso has four DSS sites located in Nouna, Ouagadougou, Oubritenga and Sapone, while Gambia has one in Farafenni [14]. The climate of both countries is similar with a short and intense rainy season occurring between June and October. Malaria transmission intensity is generally more marked in Burkina Faso[15], than in The Gambia [16].

## Methods

A literature search was done on papers containing information on mortality and malaria among children below five years of age from each country for the years since 1950. The online search was done in Medline, Embase, and Popline, using key words: "Africa, child deaths, The Gambia, Burkina Faso, malaria, *Plasmodium falciparum*, mortality, child survival and morbidity". Subsequent literature searches were accomplished by reviewing the literature cited in each of the publications obtained. In addition, research groups and experts with publications related to studies conducted in these countries were contacted for further information. Each of the papers obtained was indexed and thoroughly reviewed to extract all relevant information. The following information was obtained: study period for the data presented (the year the study started and ended), duration of the study, number of children less than five years included, overall number of deaths during the study, number of deaths due to malaria, person-years of follow-up, all-cause mortality, and malaria-specific mortality. The information in the papers was cross-checked twice to ensure that the information extracted was consistent with the information in the original paper.

### Mortality information

Mortality information was summarized as infant mortality rate (IMR: <1 years), child mortality (CMR: 1–4 years) and under-five child mortality (U5MR: 0–4 years) per 1,000 person-years. Mortality information was recorded as all-cause mortality and malaria-specific mortality for all the above age categories with the exception of studies for which such detailed information was not available.

For malaria mortality estimations, studies that were conducted among young infants only (0–3 months) were excluded because many studies have shown protection of children in this age group in malaria endemic areas through acquired immunity and other mechanisms [17]. Similarly, studies from which only seasonal rates could be obtained were also excluded. For clinical trials, mortality rates were calculated in both the intervention and control groups combined and in the control group alone.

### Person-years estimations

Where available, person-years were extracted from the publication directly. For some studies, only the number of deaths and duration of the study was available. For these, person-years (PY) were estimated as:

*PY *= N*T - *((D*/*2*)* *T)*

where ***N ***is number of children in the study, ***T ***is the duration of the study in years, and ***D ***is number of deaths during the study period. Here, the assumption was made that all children who died during the study lived on average for half of the total observation time. An overall person-year based mortality rate was computed by dividing the observed total deaths by the sum of all person-years.

#### Cause of death ascertainment

In nearly all of the studies reviewed, cause of death was ascertained by using the verbal autopsy method [18,19]. This method involves interviewing a relative of the deceased child on symptoms and signs of the illness before death. By reviewing the circumstances surrounding the time of death as described in the interviews and by studying any available clinical records, clinicians allocate a potential cause of death. Details of the verbal autopsy procedures are available elsewhere [19].

#### Mortality rate calculations

After compiling information on number of deaths and observed/estimated person years, all-cause mortality rates (MR) per 1,000 person-years were calculated as;

MR = (D/PY)* 1000

where ***D ***is number of all observed deaths during the study period and **PY **is the observed or estimated total person-years. For the malaria mortality rate, ***D ***was replaced with the observed number of all malaria-specific deaths. If applicable, mortality for the control arm was recorded separately.

For the surveys and censuses (see Table 1 and Table 2) which are cross-sectional studies, mortality information generated from collecting birth histories and child survival from mothers who were interviewed was used to estimate mortality. The mortality information was given as the probability of dying before reaching the *nth *birthday. To enable comparisons with other studies, the respective probabilities of dying in the different age-groups were converted to mortality rates per 1,000 per year using the following method [20];

**Table 1:** All-cause mortality rates in The Gambia for children under five from survey and censuses

				Mortality per 1000^2^			
					
Study type	N^1^	Survey/census	Year(s) of study	IMR	CMR	U5MR	Reference
Gambia Population census	79916 ^3^	Census	1973	243.4		67.8	[52]
	111451 ^3^		1983	182.2		59.8	
	168217 ^3^		1993	87.7		27.6	
							
Contraceptive prevalence & Fertility Determinants Survey	2521	Survey	1976–80	118.5	53.7	66.1	[27]
			1981–85	103.0	31.6	45.7	
			1986–90	93.6	20.6	34.9	
							
UNICEF, Multiple Indicator Cluster Surveys	3849	Survey	1995	100.8		29.4	[12]
			2000	96.4		27.4	
							
National Survey on Maternal mortality, Child mortality and contraceptive Prevalence	9359	Survey	2001	87.7	14.4	29.0	[53]
							
World Bank Surveys plus other National surveys	-^4^	Survey	2004	93.1		26.0	[54]

^1 ^Number of children below five years.

^2 ^IMR = Infant Mortality Rate(0–1), CMR = Child Mortality Rate(1–4 years), U5MR = Under-5 Mortality Rate(0–4) IMR is rate per 1,000 live births, CMR and U5MR are rates per 1000 person-years.

^3^Estimate of children under five years from census population

^4 ^Number based on combination of many surveys.

**Table 2:** All-cause mortality rates from Burkina Faso for children under five from surveys and censuses

				Mortality per 1,000 ^2^			
					
Study type	N^1^	Survey/census	Year(s) of study	IMR	CMR	U5MR	Reference
Burkina Faso Population census	116000^3^	Census	1975	182.2	32.8	62.2	[55]
	1476000^3^		1985	143.6	24.9	48.4	
	1975000^3^		1996	113.2	19.5	38.2	
							
Survey on mortality in Sahel(EMIS)	13421	Survey	1986	92.1			[56]
			1987	133.3			
							
Demographic and Health Surveys	5828	Survey	1992–93	98.3	28.7	45.6	[57]
	5953		1998–99	111.2	33.9	49.2	
	10645		2003	84.9	29.5	40.5	
							
UNICEF based studies including multiple Indicator cluster surveys (MICs)	-^4^	Survey	1960	199.0		74.8	[12]
			1970	177.5		67.8	
			1980	150.5		56.4	
			1990	125.4		46.9	
			1995	116.4		46.2	
			2000	113.0		46.2	
							
World Bank Surveys plus other National surveys	-^4^	Survey	2000	105.3		43.5	[54]
			2004	101.7		42.5	

^1 ^Number of children below five years.

^2 ^IMR = Infant Mortality Rate(0–1), CMR = Child Mortality Rate(1–4 years), U5MR = Under-five Mortality Rate(0–4) IMR is rate per 1,000 live births, CMR and U5MR are rates per 1000 person-years.

^3 ^Estimate of children under five year from census population.

^4^Results based on combination of many surveys.

*λ*_*x *_= *2q*_*x*_*/(2-q*_*x*_)*Δ_*x*_

where *λ*_*x *_is estimated mortality rate for the age group *x*, *q*_*x *_is the probability of dying before reaching the *n*th birthday and *Δ*_*x *_is the length in years of the age interval.

#### Modelling

To model the under five malaria and overall mortality rate *λ*_*ij *_by country (Burkina Faso: *i *= 0; Gambia: *i *= 1) and calendar year *j *(*j *= 1950 to 2004), a linear model was used as follows; *λ*_*ij *_= *α *+ *β*_1_*i *+ *β*_2 _*f(j) *+ *β*_3_*ij *with √(*PY*_*ij*_) as weights to account for study size, with different functions of *f *to investigate a possible non-linear relation between rate and calendar year, and with a factor to assess a possible interaction between country and year. All studies described in Tables 1 to 5 were used in the modelling. Since for survey/censuses the person-years were unknown, the weights were chosen equal to that of the largest cohort study. The effect of the different weights was investigated with a sensitivity analysis. For clinical trials, only results from the control arms were used.

**Table 3:** All-cause mortality rates in The Gambia for children under five from longitudinal studies

				Annual Mortality rate ^2^			
Study area	N^1^	Study type	Period of study	IMR	CMR	U5MR	References
Mandinka villages	1698	Longitudinal	1946–51	*531.6*			[30]
Keneba village	915	Longitudinal	1951–55	*242.2*	*82.8*	*112.2*	[28]
			1956–60	*212.4*	*101.3*	*120.8*	
			1961–65	*302.8*	*105.7*	*140.5*	
			1966–70	*287.0*	*124.2*	*151.3*	
			1971–75	*172.7*	*109.4*	*119.8*	
Manduar village,	441	Longitudinal	1951–55	*32.5*	*21.9*	*24.0*	[28]
			1956–60	*182.2*	*60.9*	*84.3*	
			1961–65	*154.0*	*70.1*	*86.0*	
			1966–70	*157.5*	*87.9*	*100.6*	
			1971–75	*196.6*	*71.4*	*95.4*	
Farafenni area	2505	Longitudinal	1981–82	*152.9*	*43.2*	*64.8*	[58]
	8091		1983–86	*125.4*	38.5	55.6	[34]
	2000		1983	103			[59]
	1671		1983–87			*39.7*	[42]
	1082		1982–83	*154.8*	*44.6*	*70.2*	[25]
	3194		1983–86	*134.3*	*37.3*	*58.5*	
	3206		1989–92	*74.5*	*34.6*	*43.9*	
	2270		1992–94	*90.9*	*37.7*	*49.3*	
	2161		1994–96	*78.5*	*33.7*	*43.9*	
	1364		1999			*17.7*	[60]
	487		1999			*29.0*	[61]
	17000^3^		1998–02	*84.2*	*28.4*	*40.5*	[62]
							
Upper River Division	2393	Longitudinal	1982–83	77	42.5	49.5	[63]
	931		1986–87	40	20.1	23.8	
	389		1985			*20.6*	[64]
	25670		1988–89	*96*	17.1	*35.8*	[22]
	5414		1989^4^	*92.0*	18.7	110.6	[29,65]
	5119		1990	*71.7*	16.5	88.2	
	5256		1991	*80.5*	22.1	102.6	
	5324		1992	*71.8*	15.0	86.8	
	5781		1993	*84.1*	21.3	105.4	
Lower River Division (South Bank)	3801	Longitudinal	1977	*206.9*		82.8	[31]
	3801		1982	*165.7*		60.8	
	3801		1987	*137.9*		46.8	
	20000 ^3^		1988–89	120	41	*66.0*	[21]
	1538		1988	*127.1*	31.5	*50.6*	[66]
	1592		1989	*105.1*	24.2	*41.2*	
Banjul area	9584	Hospital-based	1988–90			*35.6*	[67]
	576		1992–94			*25.2*	[23]
Several divisions	18911	Longitudinal	1992–93	72.4		*19.9*	[68]
	2025		1949–75	*221*	86	145.0	[32]
	980		1975–84	*61.7*	21	35.1	
	976		1985–97	*37.8*	8	18.0	

^1 ^Number of children below five years.

^2 ^IMR, CMR and U5MR are rates per 1,000 person-years. Estimated mortality rates are in *italics*. IMR = Infant Mortality Rate (0–1), CMR = Child Mortality Rate (1–4 years), U5MR = Under-five Mortality Rate (0–4 year).

^3 ^Whole population and not under-5 year population.

^4 ^Rates per 1,000 mid-year population

**Table 4:** Malaria mortality rates in The Gambia for children under five from DSS based studies

			Rates per 1,000 PY ^2^			
Study area	N^1^	Year(s) of study	IMR	CMR	U5MR	Reference
Farafenni area	3398	1981–83	6.3	10.7	8.5	[39]
	2505	1982–83		*13.6*	*9.9*	[58]
						
	768	1990–91			*18.2*	[69]
	651	1991		*7.9*	3.5	[68]
						
	173	1993–94		*18.5*		[70]
	487	1999			*12.5*	[61]
	1364	1999			*11.8*	[60]
						
Banjul area	9584	1988–90			*14.5 *^4^	[67]
	576	1992–94			*12.6 *^4^	[23]
						
Upper River Division	2393	1982–83		9.6		[63]
	931	1986–87		5.1		
	389	1985			*13.0*	[64]
	25670	1988–89	7.8	5.6	*6.3*	[22]
	5414	1989	10.3	6.3	*7.3*	[29,65]
	5119	1990	8.8	7.2	*7.6*	
	5256	1991	14.1	9.6	*10.6*	
	5324	1992	5.3	4.6	*4.8*	
	5781	1993	8.8	9.1	*9.0*	
	624	1992–94		*15.6*		[71]
						
Lower River Division (South Bank)	20000^3^	1988–89	13.0	*16.7*	*15.8*	[21]
	1538	1988	2.8	*11.1*	*6.9*	[66]
	1592	1989	2.8	*11.3*	*7.1*	[31]

^1 ^Number of children below five years.

^2 ^IMR, CMR and U5MR are rates per 1,000 person-years. IMR = Infant Mortality Rate(0–1), CMR = Child Mortality Rate(1–4 years), U5MR = Under-five Mortality Rate(0–4). Estimated mortality rates are in *italics*.

^3 ^Whole population and not under-5 year population.

^4 ^Malaria diagnosis laboratory confirmed.

**Table 5:** All-cause and malaria mortality rates from Burkina Faso for children under five based on DSS

				All-cause Mortality per 1,000 PY ^2^				
						
Study area	N^1^	Study type	Year(s) of study	IMR	CMR	U5MR	Malaria mortality rate <5	Reference
Kongondjan area	365	Cohort	1982–86			36.2		[72]
Pissila and Yako area	9085	Cohort	1985–96	90		49.4		[73]
								
Ouagadougou	6860	Hospital based	1993–94			*27.7*	*13.8*	[74]
								
Oubritenga province.	27577	Cohort	1993–94	102.0	36.4	44.3		[24,26]
			1994–96	101.5	30.5	39.3		
			1996–98	50.3	19.7	23.6		
			1998–00	56.7	26.1	29.9		
								
Nouna^3^	4720	Cohort	1993			*32.5*		[75]
	4786		1994			*41.6*		
	4899		1995			*35.7*		
	4840		1996			*43.1*		
	4895		1997			*30.5*		
	5323		1998			*30.9*		
								
Nouna	353	Cohort	1999			68.0	*28.3*	[8]
								
Nouna^3^	11155^4^	Cohort	1999–03	60.5	23.6	31.9	13.3	[76]

^1 ^Number of children below five years

^2 ^IMR, CMR and U5MR is per 1,000 person-years. IMR = Infant Mortality Rate(0–1), CMR = Child Mortality Rate(1–4 years), U5MR = Under-five Mortality Rate(0–4). Estimated mortality rates are in *italics*

^3 ^Results based on complete DSS analysis

^4 ^Result is 2003 mid-year population.

## Results

The literature review identified thirty nine longitudinal (clinical, epidemiological, cohort) studies from The Gambia in which malaria had been studied among children below five years of age. Twenty-one of the 39 studies had information on mortality (seventeen of these studies contained cause-specific mortality information). In addition, ten surveys and censuses were identified for which only all-cause mortality information was available.

Similarly, eighteen longitudinal studies on malaria where identified for Burkina Faso, with eight studies containing mortality information. Three of these studies contained cause-specific mortality information. In addition, fifteen surveys and censuses were identified for which all-cause mortality information was available.

In six studies (four from The Gambia, two from Burkina Faso) [21-26], a separate rate estimation for the control arm was performed.

### Trends of mortality in The Gambia

#### Mortality rates based on surveys and censuses

**Figure 1:**
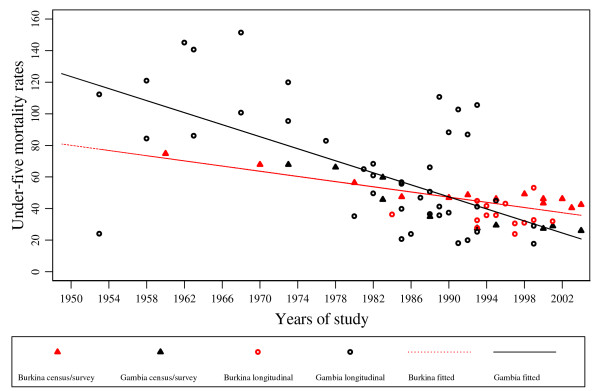
Burkina Faso and The Gambia all-cause mortality rates per 1,000 and fitted regression lines for children children under five, estimates from censuses/surveys and longitudinal studies. The black line is the fitted regression line for The Gambia and the dotted red line is the fitted regression line for Burkina Faso.

**Figure 2:**
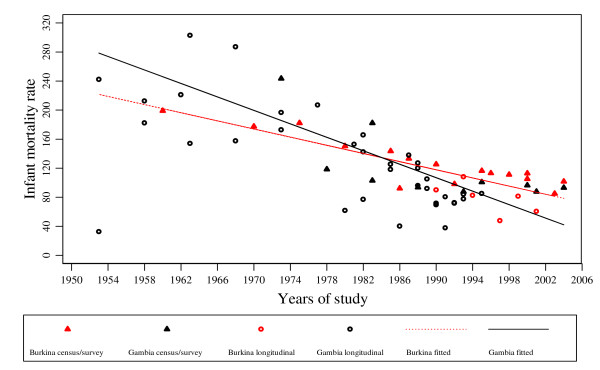
Burkina Faso and The Gambia all-cause mortality rates per 1,000 and fitted regression lines for Infants, estimates from censuses/surveys and longitudinal studies. The black line is the fitted regression line for The Gambia and the dotted red line is the fitted regression line for Burkina Faso.

Table 1 shows under-five year all-cause mortality rates for The Gambia following a review of studies based on surveys and censuses conducted between 1973 and 2004. The information is summarized as infant mortality rate (IMR), child mortality rate (CMR) and under-five mortality rate (U5MR). Two major types of survey have been conducted repeatedly (contraceptive prevalence and fertility determinants surveys and UNICEF multiple indicator cluster surveys). Three population censuses were conducted in 1973, 1983 and 1993. Mortality rates obtained from the 10-year periodical census show that IMR and U5MR declined significantly from 243 and 67.8 in 1973 to 87.7 and 27.6 in 1993 respectively (Table 1). Estimates of mortality based on surveys show similar declines in mortality, however, these are not as pronounced as census based estimates. In contrast, results reported by Pacque-Mangolis and colleagues[27] from a survey conducted in nearly the same time period provided much lower rates for IMR (Table 1). Figures 1 and 2 show clearly that mortality has declined over time.

Mortality patterns by age have also changed over time. Surveys conducted in The Gambia in the period 1976–80 showed that the IMR was about twice that of the CMR (118.5 vs. 53.7). However, for the period 1981–85 and 1986–90, the IMR was about three to four times the CMR (Table 1). A survey conducted in the year 2001 showed that the IMR was about six times the CMR (87.7 vs. 14.4).

#### Mortality rates based on longitudinal and follow-up data

Information on child mortality rates obtained from longitudinal or follow-up studies conducted in The Gambia between 1946 and 1999 is summarized in Table 3. Variations in the estimates obtained in these studies were observed, even when the reference period or year was the same. A number of studies reported mortality estimations over a period of several years [25,28,29]. In 1946, the IMR was estimated at 531.6 per 1,000 live births in three Mandinka villages in The Gambia [30]. Studies conducted in Keneba and Manduar villages [28] for the period 1951 to 1975 showed different mortality rates (sometimes with big outliers) between villages although, overall, no clear improvements in mortality were noticeable over this time period. Longitudinal studies in the same area conducted after 1975 showed a declining mortality rate [31]. Hill and colleagues[25] report similar patterns for the period 1982 to 1992 in the Farafenni areas (see Table 3). Rayco-Solon and colleagues[32] provide estimates for three-time windows between 1949 and 1997 and show clearly that mortality has declined over time in The Gambia.

Figures 1 and 2 show the distribution of all-cause mortality rates by mid-year of study based on data from longitudinal studies and surveys/censuses. Model predictions for all-cause mortality rates show that over time mortality has declined in The Gambia. Overall, the under-five all-cause mortality rate for The Gambia averaged 57.8 and 58.2 per 1,000 person-years for the period 1946 to 1999 with studies including combined arms, and with studies including control arms only, respectively.

Mortality patterns across age-classes and by time based on the results of longitudinal studies (Table 3) are similar to those observed in the studies based on surveys and censuses. For example, for the period 1949 to 1975, Rayco-Solon and colleagues [32] report an IMR which is about 2.5 times the CMR (221 vs. 86). For the subsequent periods 1975–84 and 1985–97, they estimated an IMR of about three and five times the estimated CMR respectively.

#### Malaria mortality

**Figure 3:**
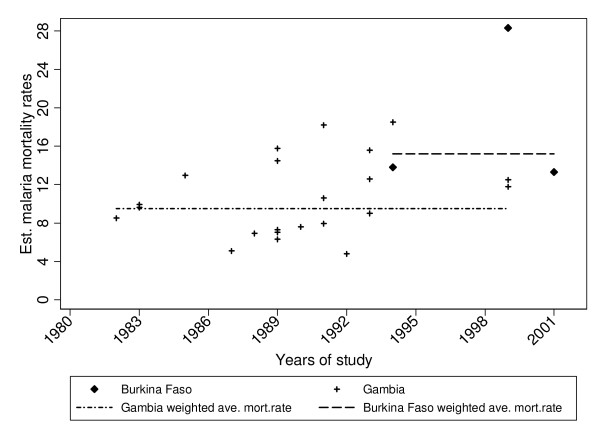
Malaria mortality rates (MMR) per 1,000 in Burkina Faso and in The Gambia for children under five, 1981–2003. The lines represent the average malaria mortality rates weighted for study sample sizes.

Malaria mortality rates show no clear change for the period 1981 to the late 1990s (see Table 4 and Figure 3). As each of the studies had different aims and designs, comparisons across studies and by regions and time (years) is difficult. For instance, in the Upper River Division, no consistent pattern can be seen and there are wide variations in rates between years. However, in the period 1988–90 the lowest malaria mortality rate (U5MR per 1,000 PY) was seen in the Upper River Division (6.3) in contrast to 18.2 in the Farafenni area, whereas results were comparable in South Bank Division (15.8) and the Banjul area (14.5).

Figure 3 shows malaria mortality rates with the respective mid-study dates. The weighted average under-five malaria-specific mortality rate for The Gambia (1981–1999) was 9.5 when all data were used and 10.1 per 1,000 person-years when clinical trial data were restricted to the control group.

### Trends of mortality in Burkina Faso

#### Mortality rates based on survey and census data

A summary of all-cause mortality rates based on surveys undertaken in Burkina Faso is given in Table 2. Overall, IMR and U5MR show a declining trend for the period 1960 to 2004 (Figure 1 and 2). Mortality rates have fallen by around a half during the period 1960 to 2004 (IMR: 199 vs 101.7 and U5MR: 74.8 vs 42.5). For surveys and censuses with results on CMR, the ratios of IMR to CMR were compared. This ratio was persistently about 5.5 and 3 times for the censuses and DHS surveys respectively.

#### Mortality rates based on longitudinal data

The results for all-cause and malaria-specific mortality rates obtained from longitudinal studies conducted in Burkina Faso are summarized in Table 5. The rates for all-cause mortality show that the U5MR has fluctuated over time with no clear trend. For example, the U5MR rates for the period 1993 to 1999 in the Nouna study region were on average between 30 and 40 per 1,000 person-years. The overall under-five all-cause mortality rate for Burkina Faso for the period 1983 to 2003 based on these studies averaged 32.7 per 1,000 person-years when all data were used and 33.6 per 1,000 person-years when clinical trial data were restricted to the control group (Table 5). These patterns for U5MR are shown in Figure 1. There are a few studies with results for IMR, but among these an overall decline in the rates for the period 1993 to 2000 can be seen (Table 5 and Figure 2).

#### Malaria mortality

The weighted malaria mortality rate for the period 1999–2003 averaged 15.4 and 15.6 per 1,000 person-years with all studies including combined arms, and with all studies including control arms only, respectively (Table 5 and Figure 3). The malaria-specific mortality rates reported here come mostly from one study site (Nouna) in Burkina Faso and are within the range of previously estimated figures from other countries in West Africa [33].

#### Comparison of Burkina Faso and The Gambia

Figure 1 graphically shows for both countries a clear reduction in overall mortality for children below five years from the year *j *= 1950 to the year *j *= 2004. The regression analysis gives the best fit with linear decline in both countries (p < 0.001), which is significantly stronger in The Gambia (*i *= 1) than in Burkina Faso (*i *= 0) (p = 0.006) with the regression equation;

*λ*_*ij *_= *80.03 *+ *43.53i *- 0.82 (j*) - 1.084ij*, j* = *j *- 1950 *(R*^2 ^= *0.48*).

Similar estimates for children below one year are shown in Figure 2. For malaria, there is no significant change in the rate over the observation period (Figure 3), however the difference between both countries is significant (p = 0.02). The estimated weighted average malaria mortality rates by country, for the observation period are displayed in Figure 3. The sensitivity analysis in which an up to tenfold weight was assigned to census/survey data to account for the larger population sizes in those studies showed only marginal variations in the model estimates.

## Discussion

This study has reviewed, summarized and compared all-cause and malaria-specific mortality rates for The Gambia and Burkina Faso. Many studies have been conducted in The Gambia to investigate different aspects of malaria (1960–2000) in contrast to Burkina Faso where only a few studies have been conducted since the late 1980s to early 2000s. Findings based on censuses, surveys and longitudinal studies show that child mortality in the Gambia has declined significantly. Reasons for this decline are likely to include socio-economic development, improved health services including the establishment of primary health care and immunisation programmes and specific intervention projects [25,29,34,35]. However, since 1990, this decline in mortality has slowed down. In parts of Africa where HIV/AIDS is a major problem, mother-to-child transmission has been a major explanation for this rebound in child mortality [36]. However, The Gambia and Burkina Faso have not been severely affected by the African HIV/AIDS epidemic and seroprevalence rates in adults remain less than 4% in both countries [37].

Estimates of mortality rates from nationally representative surveys conducted from 1960 to 2003 show that child mortality has also declined in Burkina Faso. However, this trend is not clearly discernable in the few studies for which mortality was estimated from DSS longitudinal studies because of coverage of only a few years. Nevertheless, results from DSS studies from the two countries during the period 1992 to 1999 yield very comparable estimates for under-five mortality rates, although results from surveys and censuses for under-five mortality rates are slightly different for the later years. This observation suggests the need for caution in generalising results from DSS longitudinal studies to represent national figures. Differences in observed mortality rates from nationally representative surveys could be a reflection of differences in national health policies and programmes in the two countries whereas the comparable rates obtained from the DSS based studies could be due to the effects of interventions (such as insecticide treated bednets, vaccination programmes, primary health care service delivery etc) that are routinely implemented among the DSS populations [38,39].

It is not easy to fully interpret the data provided in Figures 1, 2 and 3. However, overall, all-cause mortality rates fell in both countries during the study period covered, although some peaks occurred in the early 1990's in The Gambia. Malaria mortality rates did not show a similar decline. This could be partly due to parasite resistance to chloroquine, the first-line drug for treatment of malaria during those years, as well as to a break down in community based health services due to some under-funding by the national government during the years that followed 1990 [40-43]. There is some evidence that in The Gambia the incidence of clinical malaria is now beginning to decline (Cessay, personal communication).

Mortality differences across age groups are evident in the two countries. The observed differences in age-mortality patterns are reminiscent of mortality in other countries in SSA where the mortality rates for children in the age-group 1 and 4 have been noted to be lower than the infant mortality rate [44]. In this review, the ratio of infant mortality rate to child mortality rate has been shown to have increased over time. The overall risk of dying in the first year has declined over time, but not to the same extent as the decline in the risk of dying between the ages of one and four years. Possible explanations for these results include introduction of the expanded immunization programmes (EPI) which might have had a bigger effect on CMR than on U5MR. For instance, measles is no longer a major cause of death in children between one and four years of age in many parts of Africa where effective EPI programmes are well established [45]. Other explanations might include improvements in nutrition with deaths due to malnutrition in older children being averted [46].

The estimated malaria mortality rates of 9.5 (The Gambia) and 15.4 (Burkina Faso) per 1,000 person-years differ considerably but are within the previous estimates for SSA [47]. Numerous interventions have been undertaken in The Gambia that may have had an effect on malaria including the introduction of village-based health services and insecticide-treated bednets (ITNs) [34,43,48]. Previous studies have shown variable evidence for the link between malaria mortality rates and malaria transmission intensities [49,50]. In Burkina Faso, the annual average entomological inoculation rate (EIR) is above 80 while in The Gambia an annual average EIR is less than 50 infectious bites per person per year [16]. Therefore, differences in estimated malaria mortality rates might be a reflection of differences in malaria transmission patterns, although this is controversial [11,51].

This study has several limitations. It is possible that other relevant literature might have been left out, especially literature that was not published online or in the English language. However, it is likely that the mortality trends observed are not significantly biased due to this limitation. The fact that different studies were designed to answer a given research question makes comparison of studies difficult. The sampling methods and aims of the surveys were different and although cross-comparison of child mortality rates between studies is possible, such comparisons needs to be treated with caution. On the other hand, population censuses should provide a complete enumeration and mortality rates estimated from the census should be more realistic without major sampling errors. For instance, mortality rates resulting from a bednet intervention study cannot easily be compared to mortality rates resulting from a study that evaluated the performance of primary health care. The latter is most likely to give rates that are lower due to the direct protection provided by the mosquito net which lowers malaria transmission rates. Furthermore, the gross differences in the years covered by the malaria-related studies conducted in the two countries made comparisons of estimates difficult. In The Gambia, studies have been conducted over a longer period than in Burkina Faso where most studies were conducted after the year 1990.

Efforts were made to standardize rates by either computing person years or presenting results in the same units for the same age-group categories. For some studies it was difficult to achieve such standardized rates especially when computing malaria-specific mortality rates. In many papers, desired details were not available and it was not possible to make estimates for all age-specific mortality rates.

Despite all these limitations, this review demonstrates that with available data, mortality patterns can be compared across countries in Africa. However, caution is needed in extrapolating the results obtained in DSS surveys to give national rates. Further work will include investigating the linkage between malaria transmission parameters and child mortality. This needs to be done locally using DSS based data since estimates cannot be readily inferred from national mortality figures.

## Competing interests

The author(s) declare that they have no competing interests.

## Authors' contributions

RPN coordinated the study, performed the statistical analysis and drafted the manuscript.

HB designed, supervised and participated in coordination of the study and writing-up of the manuscript.

HR supervised the analysis and coordination of the study and participated in writing-up of the manuscript.

MJ substantially participated in the conception in particular with regard to the studies in The Gambia and writing-up of the manuscript.

AS substantially participated in the conception in particular with regard to the studies in Burkina Faso and writing-up of the manuscript.

BK substantially participated in the conception in particular with regard to the studies in Burkina Faso and writing-up of the manuscript.

BG substantially participated in the conception in particular with regard to the studies from The Gambia, in the interpretation of the results, formulating the discussion and writing-up of the manuscript.

OM substantially participated in the conception, analysis and interpretation of the results, formulating the discussion and writing-up of the manuscript.
